# Supplementary material for: Chromophore carbonyl twisting in fluorescent biosensors encodes direct readout of protein conformations with multicolor switching
Source: Commun Chem. 2023 Aug 19;6:168. doi: 10.1038/s42004-023-00982-7 (PMC10439942; doi:10.1038/s42004-023-00982-7)
Supplement: Supplementary file 2 — Supplementary Information [file 42004_2023_982_MOESM2_ESM.pdf]

SUPPLEMENTARY INFORMATION

"**Chromophore carbonyl twisting in fluorescent biosensors encodes direct readout of protein conformations with multicolor switching**"

Malin J. Allert, Shivesh Kumar, You Wang, Lorena S. Beese, Homme W. Hellinga

Contents:

Supplementary Figure 1. Emission spectra for ecGBP.16C conjugates and its mutants.

Supplementary Figure 2. Emission spectra for ecGBP.183C conjugates and its mutants.

Supplementary Figure 3. Emission spectra for ttGBP.17C conjugates and its mutants.

Supplementary Figure 4. Emission spectra for ttGBP.182C conjugates and its mutants.

Supplementary Figure 5. Glucose dependence of absorption spectra.

Supplementary Figure 6. Emission spectra for gkGBP.168C•Acrylodan.

Supplementary Figure 7. Simulated annealing omit F_O-F_C maps for ttGBP F17C•Badan.

Supplementary Figure 8. Simulated annealing omit F_O-F_C maps for ecGBP W183C•Acrylodan.

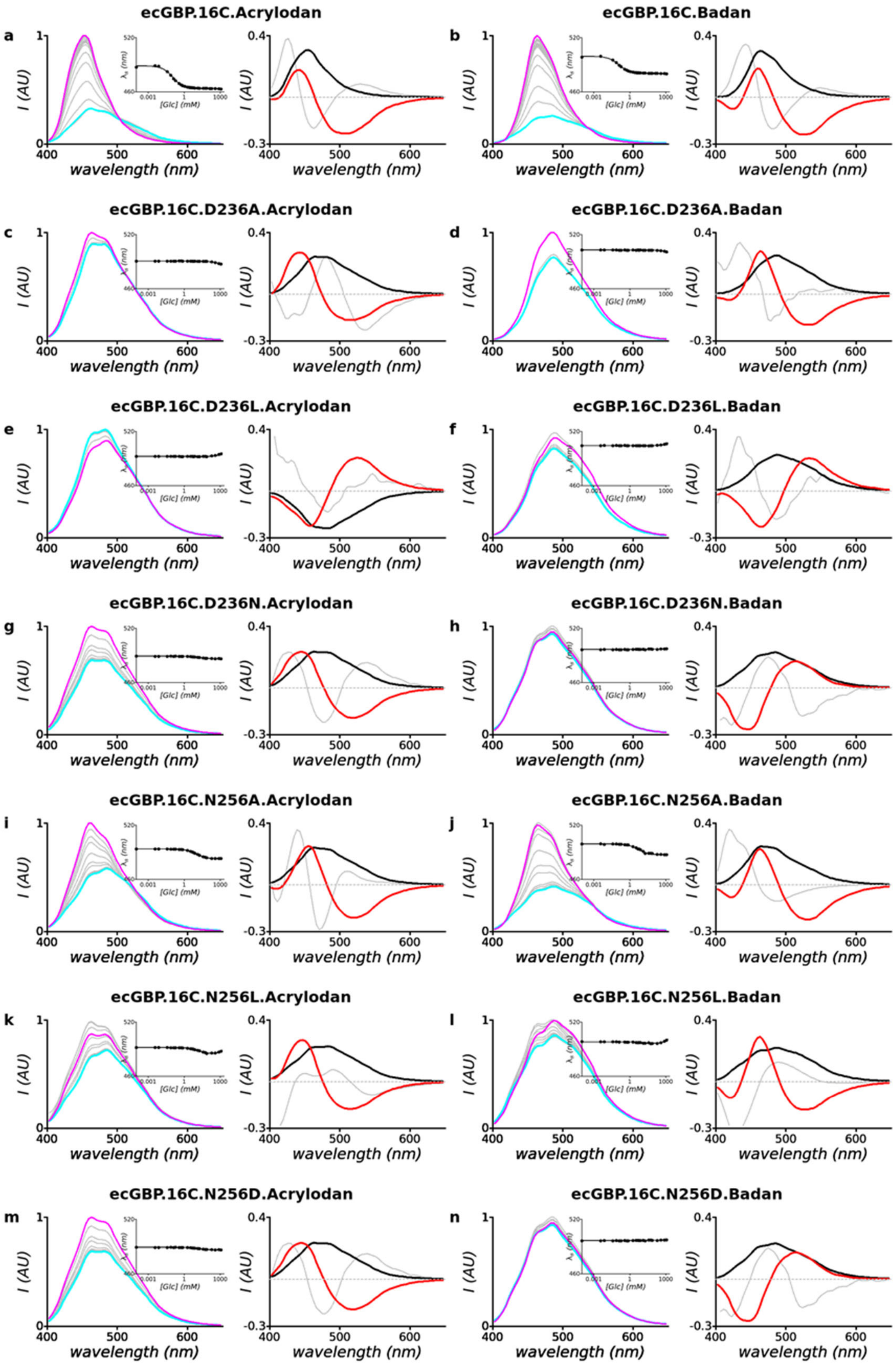
ecGBP.16C.Acrylodan
ecGBP.16C.Badan
ecGBP.16C.D236A.Acrylodan
ecGBP.16C.D236A.Badan
ecGBP.16C.D236L.Acrylodan
ecGBP.16C.D236L.Badan
ecGBP.16C.D236N.Acrylodan
ecGBP.16C.D236N.Badan
ecGBP.16C.N256A.Acrylodan
ecGBP.16C.N256A.Badan
ecGBP.16C.N256L.Acrylodan
ecGBP.16C.N256L.Badan
ecGBP.16C.N256D.Acrylodan
ecGBP.16C.N256D.Badan
a
b
c
d
e
f
g
h
i
j
k
l
m
n
I (AU)
wavelength (nm)
[Glc] (mM)

Supplementary Figure 1. Glucose dependence of emission spectra of ecGBP.16C conjugates and mutants. Each panel comprises two components; coloring and symbols as described in Fig. 2 of main text. Left, normalized emission spectra; inset, glucose titration monitored by the barychrome. Right, SVD decomposition showing the C_1 (black), C_2 (red), and C_3 (grey) spectral components.

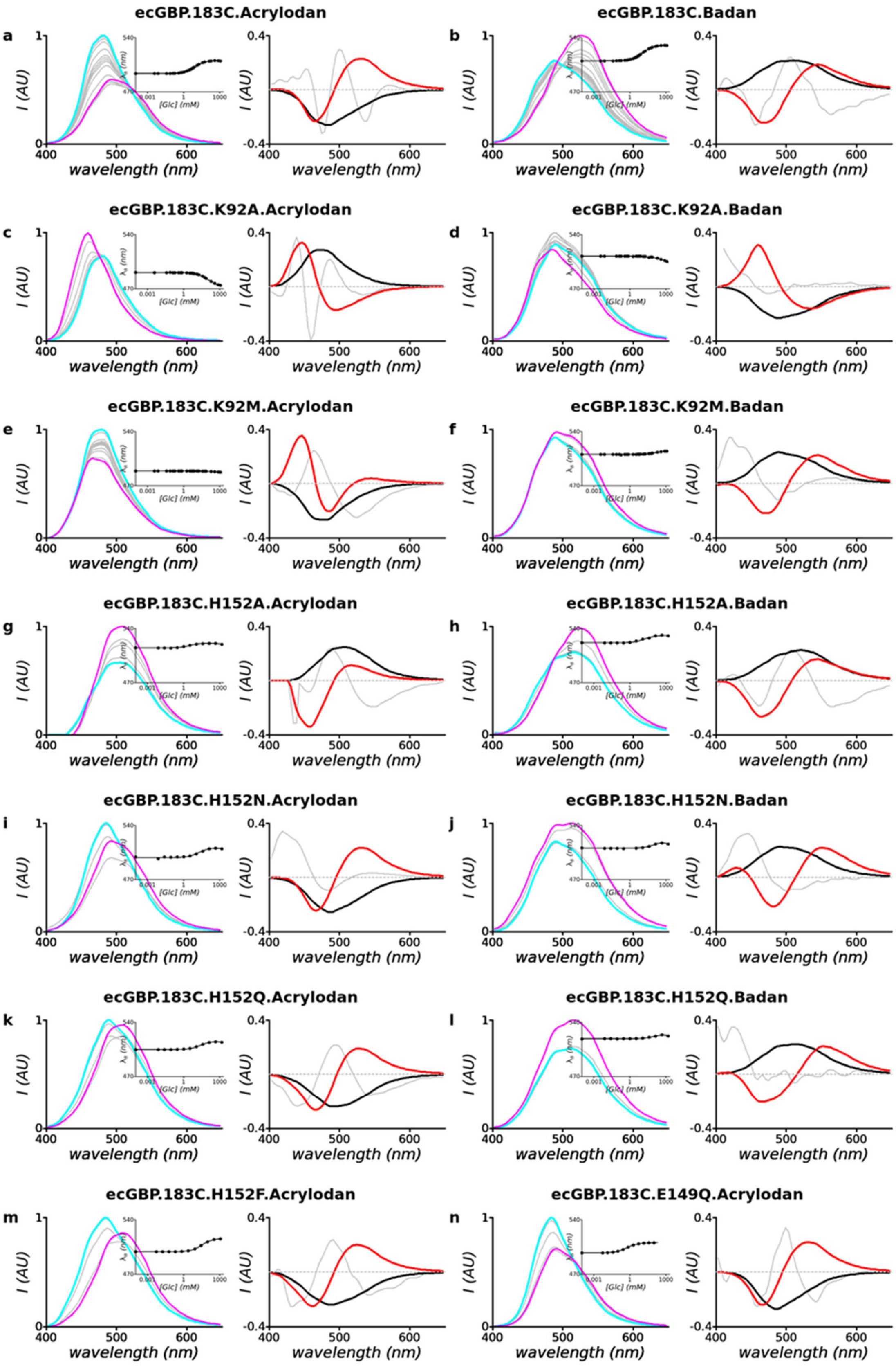
a
ecGBP.183C.Acrylodan
b
ecGBP.183C.Badan
c
ecGBP.183C.K92A.Acrylodan
d
ecGBP.183C.K92A.Badan
e
ecGBP.183C.K92M.Acrylodan
f
ecGBP.183C.K92M.Badan
g
ecGBP.183C.H152A.Acrylodan
h
ecGBP.183C.H152A.Badan
i
ecGBP.183C.H152N.Acrylodan
j
ecGBP.183C.H152N.Badan
k
ecGBP.183C.H152Q.Acrylodan
l
ecGBP.183C.H152Q.Badan
m
ecGBP.183C.H152F.Acrylodan
n
ecGBP.183C.E149Q.Acrylodan
I (AU)
wavelength (nm)
400
500
600
0
1
0.4
-0.4
[Glc] (mM)
0.001
1
1000
470
540

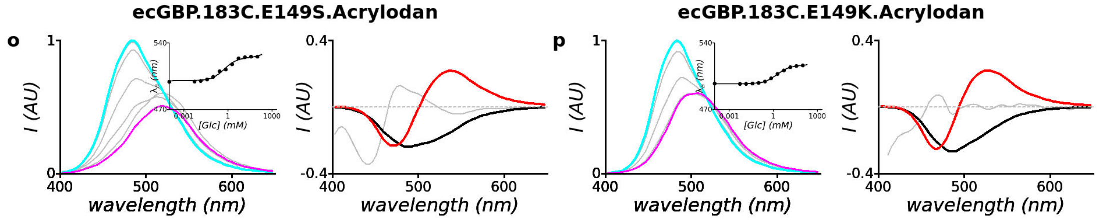

Supplementary Figure 2. Glucose dependence of emission spectra of ecGBP.183C conjugates and mutants. Each panel comprises two components; coloring and symbols as described in Fig. 2 of main text. Left, normalized emission spectra; inset, glucose titration monitored by the barychrome. Right, SVD decomposition showing the C_1 (black), C_2 (red), and C_3 (grey) spectral components.

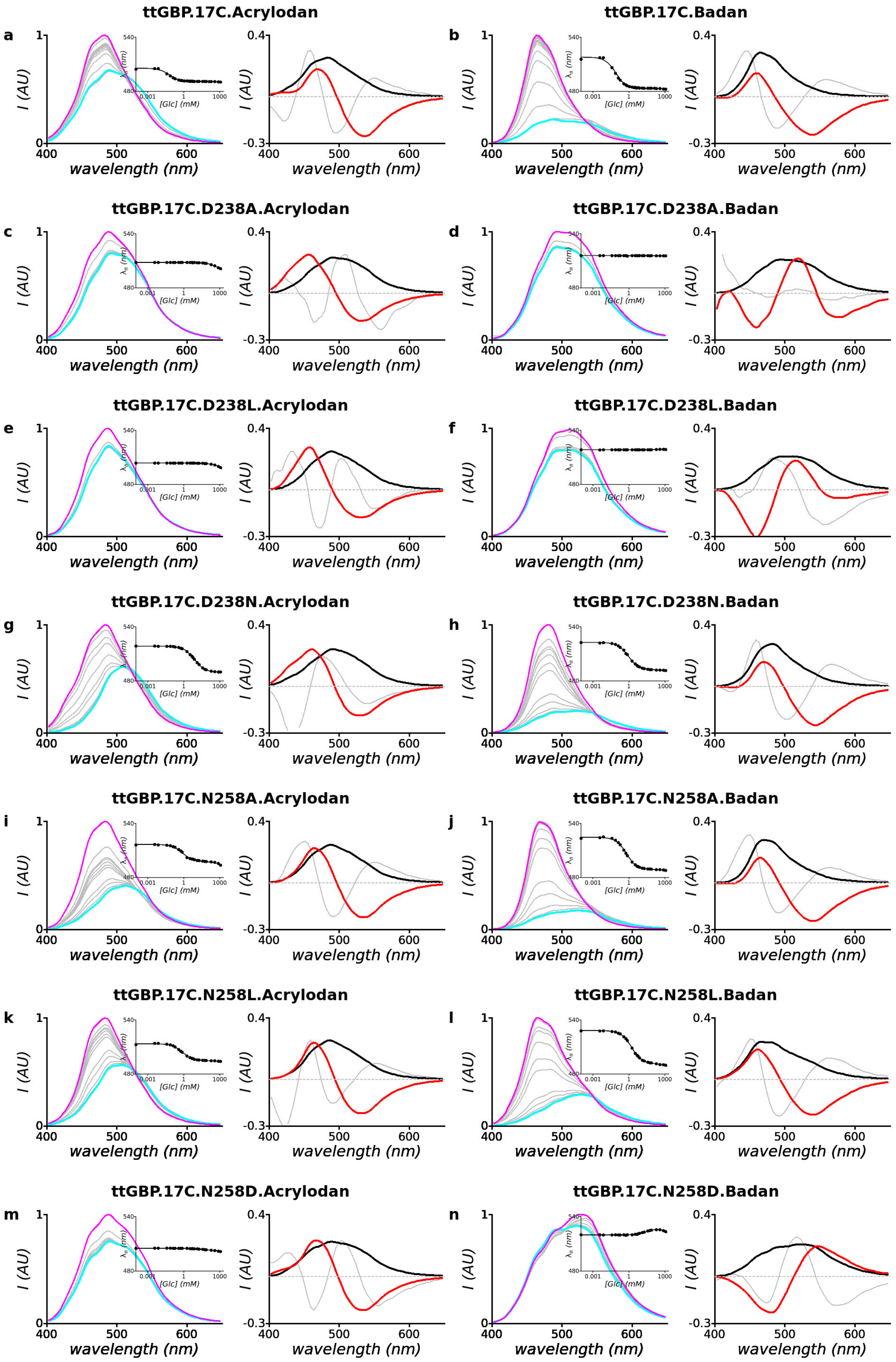

Supplementary Figure 3. Glucose dependence of emission spectra of ttGBP.17C conjugates and mutants. Each panel comprises two components; coloring and symbols as described in Fig. 2 of main

text. Left, normalized emission spectra; inset, glucose titration monitored by the barychrome. Right, SVD decomposition showing the C_1 (black), C_2 (red), and C_3 (grey) spectral components.

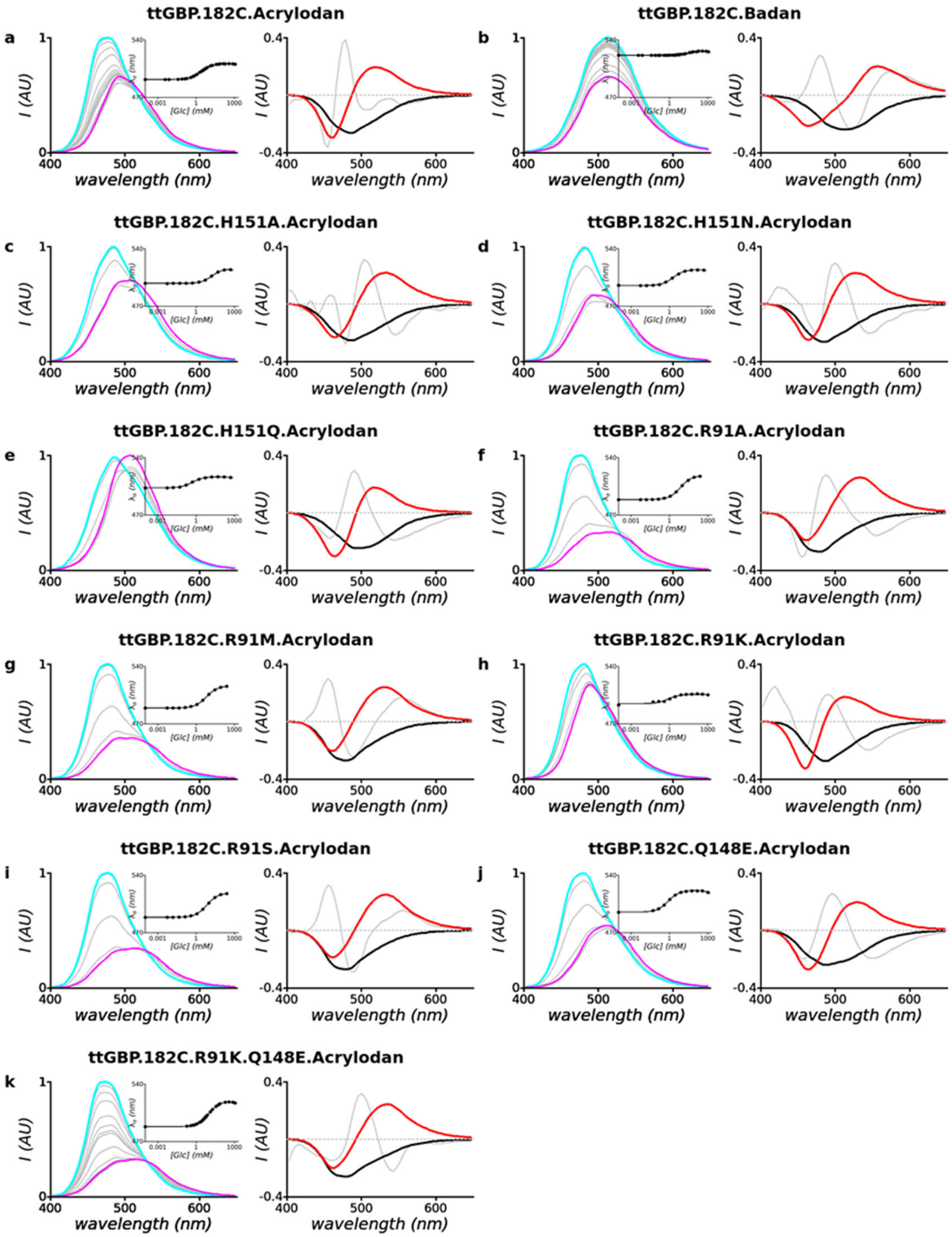

Supplementary Figure 4. Glucose dependence of emission spectra of ttGBP.182C conjugates and mutants. Each panel comprises two components; coloring and symbols as described in Fig. 2 of main text. Left, normalized emission spectra; inset, glucose titration monitored by the barychrome. Right, SVD decomposition showing the C_1 (black), C_2 (red), and C_3 (grey) spectral components.

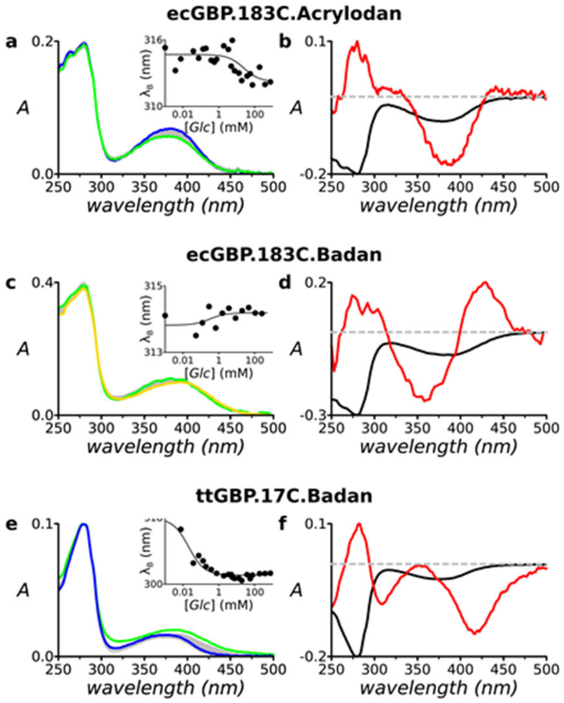

Supplementary Figure 5. Glucose dependence of absorption spectra. Left, absorption spectra (line colors indicate approximate color of spectrum: blue, green, yellow in the glucose-free and -bound forms; grey lines, intermediate glucose concentrations); inset, glucose titration monitored by the barychrome. Right, SVD decomposition showing the C_1 (black) and C_2 (red) spectral components.

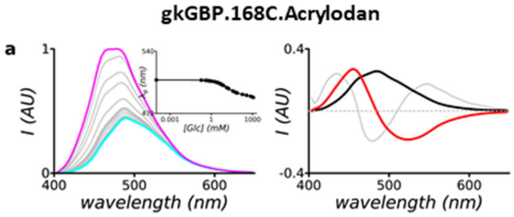

Supplementary Figure 6. Glucose dependence of emission spectra of gkGBP.168C•Acrylodan. Left, normalized emission spectra; inset, glucose titration monitored by barychrome. Right, SVD decomposition showing the C_1 (black), C_2 (red), and C_3 (grey) spectral components.

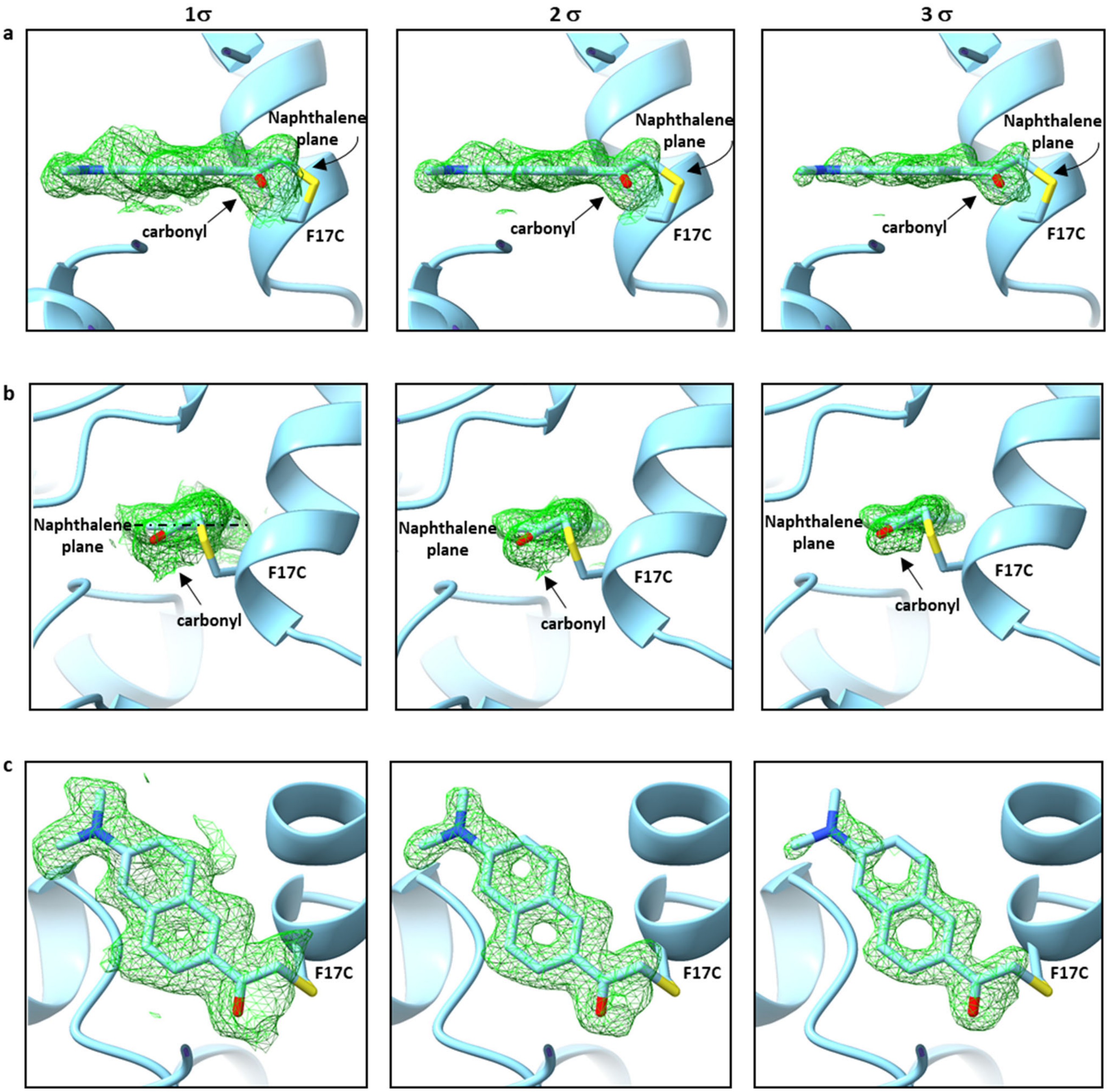

Supplementary Figure 7. Simulated annealing omit F_O-F_C maps contoured at 1 σ (left panel), 2 σ (middle panel) and 3 σ (right panel) for ttGBP F17C•Badan showing (a) the long side of the naphthalene ring, (b) the back view, and (c) the top view of the Badan conjugate. Shown is the positive density 2 Å around the Badan conjugate.

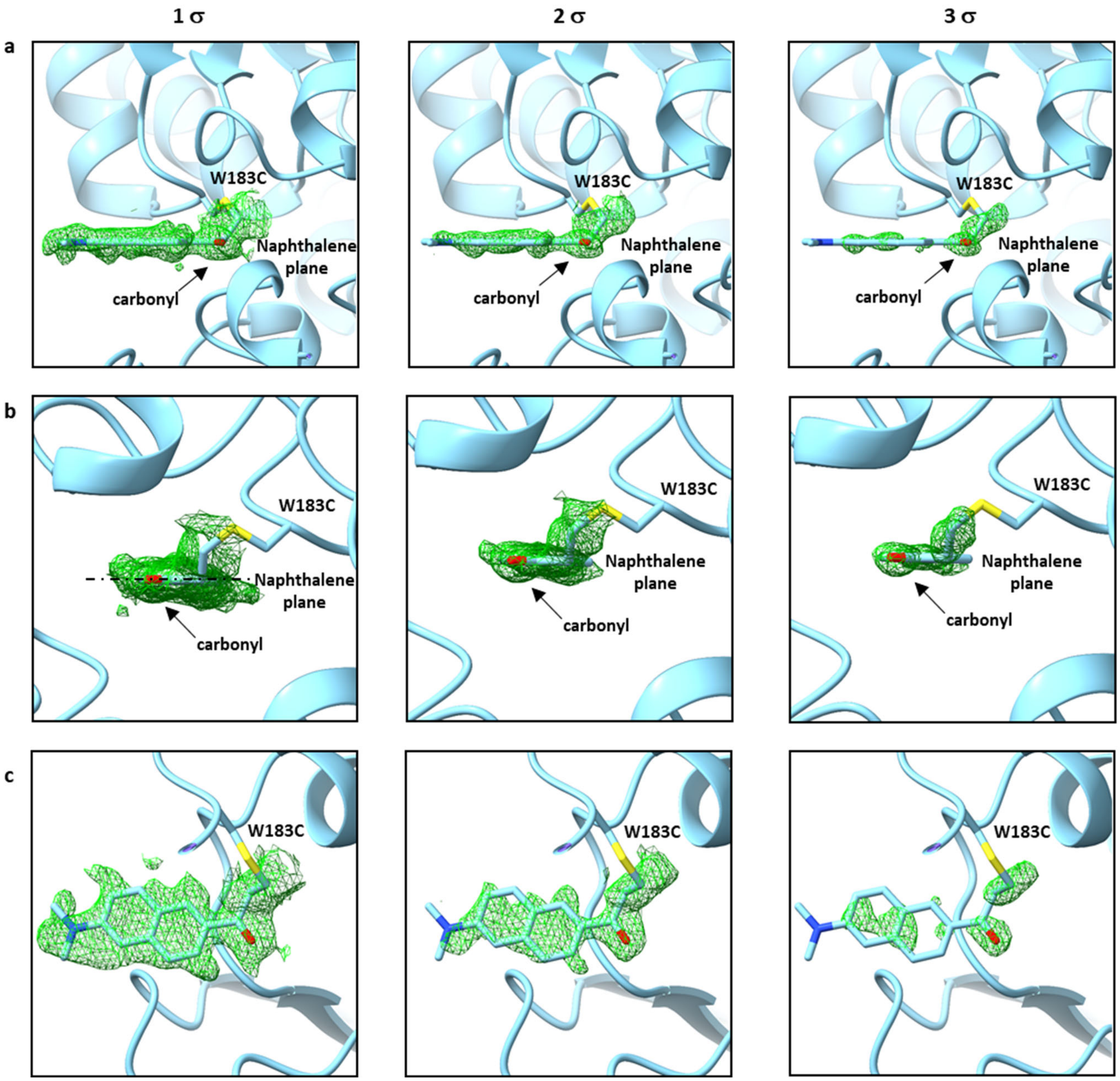

Supplementary Figure 8. Simulated annealing omit F_O-F_C maps contoured at 1 σ (left panel), 2 σ (middle panel) and 3 σ (right panel) for ecGBP W183C•Acrylodan showing (a) the long side of the naphthalene ring, (b) the back view, and (c) the top view of the Acrylodan conjugate. Shown is the positive density 2 Å around the Acrylodan conjugate.
