# Supplementary material for: Chromophore carbonyl twisting in fluorescent biosensors encodes direct readout of protein conformations with multicolor switching
Source: Commun Chem. 2023 Aug 19;6:168. doi: 10.1038/s42004-023-00982-7 (PMC10439942; doi:10.1038/s42004-023-00982-7)
Supplement: Supplementary file 3 — Description of Additional Supplementary Files [file 42004_2023_982_MOESM3_ESM.pdf]

Description of Additional Supplementary Files

File name: Supplementary Data 1

Description: Structure of ttGBP.17C.Badan

File name: Supplementary Data 2

Description: Structure of ecGBP.183C.Acrylodan
